# Community-based Rehabilitation Training after stroke: protocol of a pilot randomised controlled trial (ReTrain)

**DOI:** 10.1136/bmjopen-2016-012375

**Published:** 2016-10-03

**Authors:** Sarah G Dean, Leon Poltawski, Anne Forster, Rod S Taylor, Anne Spencer, Martin James, Rhoda Allison, Shirley Stevens, Meriel Norris, Anthony I Shepherd, Raff Calitri

**Affiliations:** 1 University of Exeter Medical School & PenCLAHRC, Exeter, UK; 2 ResearchAcademic Unit of Elderly Care and Rehabilitation, University of Leeds, Bradford, UK; 3 Royal Devon & Exeter Hospital, Exeter, UK; 4 Torbay and South Devon NHS Foundation Trust, Torquay, UK; 5 Department of Clinical Sciences, Brunel University London, Middlesex, UK; 6 Department of Sport and Exercise Science, University of Portsmouth, Portsmouth, UK

**Keywords:** REHABILITATION MEDICINE

## Abstract

**Introduction:**

The Rehabilitation Training (ReTrain) intervention aims to improve functional mobility, adherence to poststroke exercise guidelines and quality of life for people after stroke. A definitive randomised controlled trial (RCT) is required to assess the clinical and cost-effectiveness of ReTrain, which is based on Action for Rehabilitation from Neurological Injury (ARNI). The purpose of this pilot study is to assess the feasibility of such a definitive trial and inform its design.

**Methods and analysis:**

A 2-group, assessor-blinded, randomised controlled external pilot trial with parallel mixed-methods process evaluation and economic evaluation. 48 participants discharged from clinical rehabilitation despite residual physical disability will be individually randomised 1:1 to ReTrain (25 sessions) or control (exercise advice booklet). Outcome assessment at baseline, 6 and 9 months include Rivermead Mobility Index; Timed Up and Go Test; modified Patient-Specific Functional Scale; 7-day accelerometry; Stroke Self-efficacy Questionnaire, exercise diary, Fatigue Assessment Scale, exercise beliefs and self-efficacy questionnaires, SF-12, EQ-5D-5L, Stroke Quality of Life, Carer Burden Index and Service Receipt Inventory. Feasibility, acceptability and process outcomes include recruitment and retention rates; with measurement burden and trial experiences being explored in qualitative interviews (20 participants, 3 intervention providers). Analyses include descriptive statistics, with 95% CI where appropriate; qualitative themes; intervention fidelity from videos and session checklists; rehearsal of health economic analysis.

**Ethics and dissemination:**

National Health Service (NHS) National Research Ethics Service approval granted in April 2015; recruitment started in June. Preliminary studies suggested low risk of serious adverse events; however (minor) falls, transitory muscle soreness and high levels of postexercise fatigue are expected. Outputs include pilot data to inform whether to proceed to a definitive RCT and support a funding application; finalised Trainer and Intervention Delivery manuals for multicentre replication of ReTrain; presentations at conferences, public involvement events; internationally recognised peer-reviewed journal publications, open access sources and media releases.

**Trial registration number:**

NCT02429180; Pre-results.

**Strengths and limitations of this study**

- This pilot randomised controlled trial study meets the Medical Research Council (MRC) guidance on the development and evaluation of complex interventions and includes comprehensive patient and public involvement.
- This preliminary evaluation of a late stage rehabilitation programme addresses the gap in the evidence related to what facilitates stroke recovery in the longer term.
- This small scale study is designed to estimate effect sizes but has insufficient statistical power to detect differences in outcomes between groups.
- The follow-up period is relatively short compared with what would be planned for a fully funded definitive trial.

## Introduction

Residual physical disability is common following discharge from stroke rehabilitation services. A third of first-time stroke survivors remain physically disabled 5 years after their stroke,^1^ equivalent to more than 300 000 people in the UK (ref. 2, p. 51). Stroke services are traditionally ‘front loaded’ with provision tailing off a few months after stroke.^3^ However, people with stroke report a variety of unmet long-term needs and a sense of being abandoned by National Health Services (NHS).^4^ The England National Stroke Strategy recommends that stroke be regarded as a long-term condition and that continuing support is provided for those who need it.^5^ This includes community-based rehabilitation, with an emphasis on personalisation, reablement and self-management of the consequences from stroke.^5^ There is good evidence that exercise can promote functional recovery,^3^ enhance adjustment and coping,^6^ improve psychological well-being,^6^ and reduce the risk of recurrence.^7^ Hence, stroke guidelines recommend that people with stroke should regularly engage in specific forms of exercise;^8^ ^9^ however, many individuals do not meet these recommendations.^10^ ^11^ Various personal and environmental factors may account for this: stroke-related impairments, lack of confidence or knowledge regarding exercise and its benefits, and inadequate provision of support programmes and facilities. In response, community-based programmes are being offered;^12–14^ however, these programmes often focus on fitness rather than function, giving little attention to self-management or to sustaining behaviour (to ensure benefits are maintained after structured programmes have ended). National stroke guidelines^8^ recommend interventions address functional improvement^15^ and self-management^16^ strategies even though a recently updated Cochrane review^17^ notes the gap in evidence regarding these interventions.

An approach called Action for Rehabilitation from Neurological Injury (ARNI) attempts to address these concerns; it was created specifically for people with stroke and acquired brain injury who wish to continue their functional recovery.^18^ ARNI is not a rigidly defined programme but a set of principles and strategies tailored to individual circumstances and contexts. It is led by registered exercise professionals who have been additionally trained and accredited by the ARNI Institute (http://www.arni.uk.com). Our research into ARNI started because a stroke survivor participating in PenCLAHRC's (http://clahrc-peninsula.nihr.ac.uk/) research question generation process asked if ARNI worked but, as yet, there have been no randomised controlled trials (RCTs) of this intervention. In the UK, the NHS, some local authorities and other organisations are using ARNI trainers to provide community-based training for stroke survivors. We surveyed known providers of this training including those located in Northeast England, Lancashire, Luton and Bedfordshire, Milton Keynes, Hillingdon and Cornwall. The survey found that training has been very positively received by stroke survivors, their families and clinicians but it varied in content and delivery (unpublished report: Poltawski, 2011). Reports of benefits by the broadcaster Andrew Marr have also increased public awareness of ARNI.^19^ However, the evidence for ARNI remains largely anecdotal, it may only work for a selected few and the approach is difficult to reproduce. There is a need for a more detailed cohesive specification of ARNI that could be rigorously evaluated and replicated. Thus, we have followed the Medical Research Council's framework for the development and evaluation of complex interventions^20^ and have undertaken five linked preliminary studies: (1) the aforementioned survey of current ARNI provision in the UK, (2) a comparison of the ARNI approach with relevant stroke practice guidelines,^9^ (3) before-and-after studies of group-based^21^ and (4) one-to-one^22^ training, and (5) focus groups conducted with our participants.^23^ From this work we have designed a programme called Rehabilitation Training (ReTrain) which is based on core ARNI principles and informed by best practice guidelines for stroke.^9^ Before undertaking a large definitive RCT of ReTrain a pilot study is needed to address issues of feasibility and acceptability.

### Aims

To undertake a study that will evaluate the feasibility and acceptability of procedures to inform the design and delivery of a definitive RCT of ReTrain (which would assess the clinical and cost-effectiveness of ReTrain for stroke survivors). The study objectives are to: (1) assess the feasibility and acceptability of recruitment, randomisation, allocation concealment and outcome assessment blinding procedures; (2) obtain an estimate of the outcome variance and retention rates to inform sample size calculations for a fully powered trial; (3) confirm the feasibility of ReTrain, its acceptability to participants and finalise the ReTrain Trainer and Intervention Delivery manuals; (4) assess outcome measurement burden for participants to confirm that data can be collected (including safety data), measures will be completed and to inform selection of primary and secondary measures for the definitive trial; (5) rehearse process evaluation methods for the definitive trial, including assessment of intervention fidelity (ie, adherence to the intervention manual by participants and trainers); (6) evaluate resource use, including carer support, and costs associated with intervention delivery, assess the feasibility of collecting health and social service resource use and explore the relative strengths of measures uses to calculate health-related quality of life (quality-adjusted life years, QALYs) and provide an economic evaluation framework for the definitive trial.

## Methods and analysis

### Design

**Figure 1:**
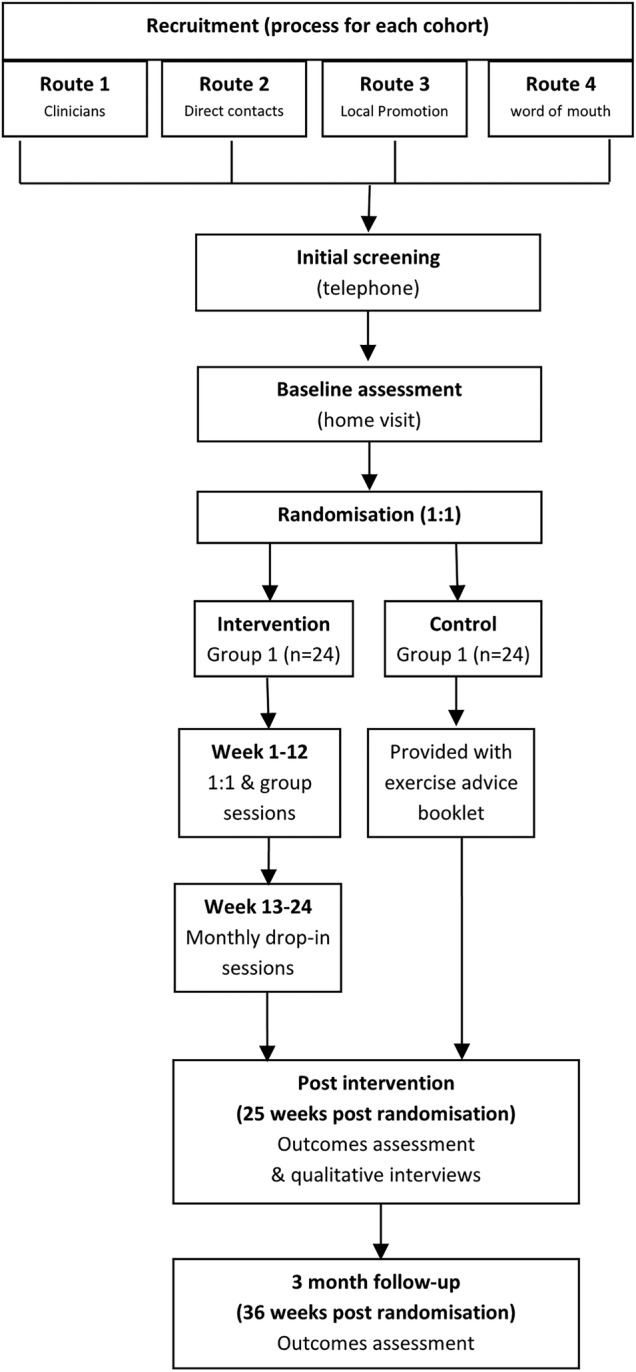
Study flow chart.

A two-group, assessor-blinded, randomised controlled external pilot trial with parallel mixed-methods process evaluation and economic evaluation. Eligible participants will be individually randomised 1:1 to intervention (ReTrain) or control (exercise advice booklet).^24^ The design is depicted in figure 1, which shows the flow of one cohort of participants (the study comprises three programmes).

#### Population

Potential participants will be included if they meet the following inclusion and exclusion criteria: they have (1) a primary clinical diagnosis of stroke (assessed by referring clinician/general practitioner (GP) records), (2) they are at least 1 month (but no upper limit) since discharge from NHS physical rehabilitation services at randomisation, (3) they are able to walk independently indoors with or without mobility aids, but have self-reported difficulty with or require help on stairs, slopes or uneven surfaces, (4) a willingness to be randomised to either control or ReTrain and to attend the training venue, (5) have cognitive capacity and communication ability sufficient to participate in the study (assessed by recruiting team using standard tools).^25^

Criterion (3) has been selected pragmatically to maximise eligibility while ensuring participants have a mobility deficit that could be addressed by the intervention. Eligible people with aphasia will not be excluded.

Potential participants will be excluded if they are <18 years old, currently (or within 1 month of) receiving ARNI-based training or have contraindications to moderate-to-vigorous physical activity (adapted from American College of Sports Medicine (ACSM) guidelines)^26^ including:

- Acute or uncontrolled heart failure;
- Unstable or uncontrolled angina;
- Uncontrolled cardiac dysrhythmia causing symptoms or haemodynamic compromise;
- Symptomatic severe aortic stenosis;
- Current deep vein thrombosis, pulmonary embolus or pulmonary infarction;
- Acute myocarditis or pericarditis;
- Suspected or known dissecting aneurysm;
- Unstable/uncontrolled blood pressure;
- Systolic blood pressure >160;
- Diastolic blood pressure >100;
- Acute systemic infection;
- Uncontrolled diabetes.

#### Sample size

The target recruitment number is 48 participants (24 per group). This number is based on the recommendation of 30 complete data sets for pilot studies in order to estimate outcome variance^27^ and running the intervention three times (Exeter I and South Devon, then Exeter II, ie, 3×8 patients) to enable investigation of variations in context and other process variables. This recruited number also allows estimation of a predicted attrition rate of 20% with a precision of ±5% with 95% certainty.

#### Participant recruitment

Participants will be recruited and the programme delivered in two areas of Devon (Exeter and South Devon). These areas cover a population of 250 000 with at least 3000 stroke survivors who require the help of others in everyday activities.^11^ To maximise potential recruitment, several routes for recruitment will be used to reach both those who are just leaving rehabilitation services and those who have been discharged for some time:

1. Via clinicians in NHS primary care, hospital and community stroke services:
  - Early supported discharge teams;
  - Community rehabilitation teams;
  - Physiotherapy outpatients departments;
  - Those responsible for conducting the 6-month review recommended by the National Stroke Strategy;
  - GP surgeries via the local Clinical Research Network (CRN).

Clinicians will provide potential participants with an invitation letter and brief description of the study and, if they are interested, obtain permission to pass on their details to the study team. The clinician will ask the potential participant to complete a short form (or complete the form on their behalf), recording their contact details and consenting for these details to be passed to the research team.

- Direct (using contacts)
  The CRN (formerly South West Stroke Research Network (SW SRN)) using hospital-based recruitment team, newsletters and targeted mailing;
  Exeter Clinical Research Facility which maintains a database of health research-interested members of the public;
  Letters, articles and posters will be used to provide brief details of the study and invite expressions of interest to contact the research team by telephone call, email or post.
- Promotion via local stroke support networks identified through national organisations such as the Stroke Association, Different Strokes and Connect, and via internet searches and in local media.
- Word of mouth, study flyers, adverts and information sheets.

We have discussed recruitment plans with two local community rehabilitation team leads and the CRN (who have checked three GP surgery's databases); from this we estimate a recruitment rate of 5–6 participants per month although this is one of the feasibility objectives being tested in this study. Our preliminary Devon-based studies recruited 2–4 participants per month, but only from the Exeter area, without formal network support, using fewer routes, and based on narrower inclusion criteria to the current proposal.

A potential issue in recruitment is the likelihood of attracting people who are already committed to exercise and not those who are currently inactive, who may be put off by the emphasis on the term ‘exercise’. Focus groups conducted previously by the research team, and our Service User Group (SUG), have suggested that promotion should emphasise the potential functional and quality of life benefits, and not promote it primarily as physical exercises. To this end, a member of the research team will brief and support the personnel involved in providing initial information about the study to potential participants, and where necessary spend time in contact with potential participants to help their understanding of the intervention and study. For those who are already committed to exercising, they will still be eligible providing they are no longer involved in a formal rehabilitation programme. Because of uncertainty in recruitment rates and patterns and the need to have eight people ready to start the training programme, some participants may have to wait several weeks until sufficient group members have been recruited. To help mitigate the potential problem of excluding those who do not live close to a venue, reasonable travel costs (eg, mileage claims, local bus and train journeys, and specialised wheelchair taxis) to and from the training venue will be offered to all participants.

#### Randomisation and group allocation

To ensure allocation concealment, participants will be allocated 1:1 to either intervention or control arms using a web-based randomisation service supported by the Peninsula Clinical Trials Unit (PenCTU). We will use minimisation procedures to ensure balance between groups on two variables: time since stroke (≤3 vs >3 months), since spontaneous recovery might be more likely among those whose stroke was relatively recent,^28^ and level of functional disability (modified Rankin Scale^29^ (mRS) score ≤2 vs >2), since this may limit the extent and nature of training possible for a participant. Once the remote randomisation service has registered and randomised the participant, allocation will be made known to the trial manager, who will not be involved in assessing patient outcomes. Following randomisation, the trial manager will contact participants to inform them of group allocation.

#### Blinding

Participants, personal trainers providing the intervention, and researchers conducting the process and economic evaluation cannot be blinded to allocation. However, outcomes will be assessed by an independent assessor blinded to group allocation. Participants, who have been informed of their allocation, will be reminded to hide their allocation from the assessor. Any incidents of unblinding will be recorded, and the assessor will be asked to record their guess of participant allocation after undertaking the assessments. Following recommended strategies to maintain and assess blinding,^30^ the outcomes assessor will not be based at the research centre.

#### Intervention

**Figure 2:**
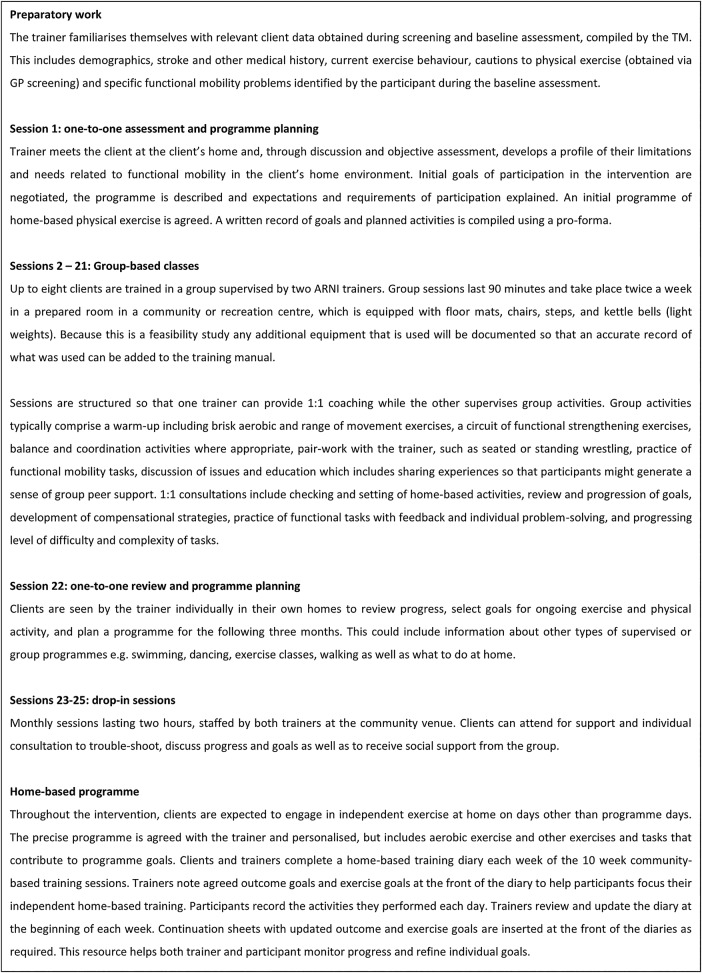
The ReTrain programme. (ARNI, Action for Rehabilitation from Neurological Injury; GP, general practitioner; TM, trial manager).

ReTrain is a specified intervention that is based on ARNI but also draws on poststroke exercise guidelines,^9^ our preliminary studies and stakeholder consultations; this combination makes it a novel intervention compared with other community exercise-based programmes. ReTrain aims to (1) enhance function through (A) task-related practice, (B) teaching adapted or compensatory strategies and (C) provide targeted strength training (cardiovascular physical fitness gains also occur through these activities); (2) develop self-management skills for ongoing rehabilitation; (3) personalise training using negotiated goals; and (4) instil a commitment to and habit of regular exercise for health improvement and longer term maintenance (once structured training programme has been completed). ReTrain focuses on functional mobility which includes safe and efficient walking in varied terrains, kerbs, cambers and in crowds, turning and moving quickly, climbing steps and stairs without rails, getting to and from the floor without furniture or other aids, and moving about without mobility aids or while carrying loads. Training will be based on an Intervention Delivery manual and led by personal trainers on the Register of Exercise Professionals (level 3 or above) who are ARNI-trained and accredited (and will be aware if there are stroke-related concerns that require referral) and they will have received additional training in the delivery of ReTrain. There will be a maximum ratio of one trainer to four stroke survivors. The use of personal trainers rather than clinicians is based on a principle of de-medicalising the rehabilitation process^18^ ^31^ ^32^ and received strong support from our SUG. It may also have cost-effectiveness benefits.^14^ ^33^ ReTrain is predominantly based in a community centre with twice weekly, 90 min sessions over 3 months, comprising: (1) an introductory one-to-one session (as a home visit); (2) group classes with up to two trainers and eight clients (preserving the maximum ratio of one trainer to four participants); (3) a closing one-to-one session (as a home visit). This is followed by three drop-in sessions over the subsequent 3 months at the community centre. The final one-to-one session and monthly drop-in sessions address concerns raised by our preliminary study participants of the sudden cessation of support.^23^ A more thorough description of the intervention can be seen in figure 2. The ReTrain Trainer and Intervention Delivery manuals were designed using an overarching theoretical framework that enabled existing evidence-based components (from the physiology of exercise training, Behaviour Change Techniques^34^ and stroke rehabilitation guidelines) to be mapped together with the as yet unresearched ARNI principles and techniques. The theoretical framework is known as the Information-Motivation-Behavioural Skills model^35^ and enables intervention mapping to take place.^36^ This mapping process will be developed and refined as part of the feasibility work of this study and will specify the essential resources, activities and behaviours of trainers and clients that must be present in sessions and across the programme. Session checklists will capture the main content and indicate where flexibility is permitted (for individualisation of training) and allow evaluation of intervention fidelity.

##### Links between ARNI and ReTrain

The ARNI principles were identified through analysis of ARNI documentation,^18^ discussion with its developer and the survey of existing ARNI programmes. Although ARNI aims to address all aspects of function, the ReTrain programme focuses on functional mobility because of its association with independence in activities of daily living and social participation. ARNI provides strategies for developing these skills that have been adapted for people with hemiplegia and other stroke-related impairments. ReTrain will not focus on upper limb function except in the service of such mobility tasks.

ARNI aims to enhance individual's ability to manage their own rehabilitation and exercise programme. This is achieved through a variety of strategies and techniques including education in exercise principles after stroke, developing skills in goal setting, functional problem solving and self-monitoring, and establishing a programme of regular independent home-based exercise. ReTrain will ensure that these activities are incorporated into the training process.

ARNI is designed as a personalised programme with substantial one-to-one training to ensure individual tailoring of activities, feedback and progression, and encouragement to work at the edge of personal capacity. The ReTrain programme combines these features with the benefits of a group programme which include peer support, pair work and efficient use of resources.^22^ ^23^ The structure of the ReTrain programme is described in figure 2, and a more detailed Intervention Delivery manual will be developed and refined as part of the study.

#### Control

All participants receive treatment as usual (which will be recorded for both groups) however, in addition, the control group will receive an advice booklet about exercise after stroke, based on a Stroke Association's publication.^24^ This offers a low-cost alternative to ReTrain that might be provided to stroke survivors, although it does not give advice on specific exercises. We believe offering an advice booklet to the control patients, will improve recruitment and retention.

#### Assessment and outcomes

##### Initial screening assessment

Those expressing an interest in taking part in the study will be contacted by telephone by a member of the research team and, with their permission, be asked questions to assess their eligibility. These will address: diagnosis of stroke, time since stroke, status regarding receipt of poststroke physical rehabilitation, mobility, potential contraindications to exercise and willingness to exercise regularly as part of intervention but also willingness to be in the control group. Permission will be sought to contact the person's GP (and/or referring clinician, if this is the recruitment route) to obtain relevant medical history and screen for contraindications. This verbal consent will be documented. A medical screening questionnaire will be sent to the GP if the person appears eligible for inclusion in the study. The research team will await receipt of the completed GP letter but will contact the practice after 7 days if there has been no reply from the GP. Once the GP letter has been received by the research team, those potentially eligible will be contacted to arrange a home-based visit. Those not eligible will be thanked and informed of the ineligibility.

##### Home-based screening and data collection

Those apparently eligible for inclusion will be visited at home by a member of the research team to provide further information about the intervention and study, and to obtain written consent. There are discrete activities within the trial that require specific consent from the participant. As part of the consent process participants will be asked whether they would be willing for a researcher to look at their medical records to view the number of primary and secondary care resources they used over the course of the trial. They will also be asked whether they would like to take part in an interview at the end of the trial to discuss their experiences. Participants will also be asked whether they would be happy to be video-recorded during some training sessions, should they be allocated to the ReTrain programme. All participants will be asked their consent again immediately prior to these activities so that they can confirm or decline consent.

Demographic and other personal information will also be collected at this time:

- Date of birth, gender;
- Stroke history (type, severity, date, summary of rehabilitation);
- mRS score, established by a simple questionnaire;^29^
- Medical history (comorbidities, current treatments);
- Social history (employment status, previous employment);
- Physical exercise history (prestroke, poststroke, last month: type, frequency, intensity).

Depending on recruitment patterns, those eligible and consenting may be required to wait several weeks before start of the intervention. They will be kept informed of timings by a member of the research team.

##### Clinical outcomes

We will collect both the objective physical outcome and participant-reported outcome measures (PROMs) that we intend to collect in a definitive trial. Outcome selection was informed by discussions with our SUG and data from our preliminary studies. The measures are: the Rivermead Mobility Index;^37^ ^38^ the Timed Up and Go Test;^39^ a modified Patient-Specific Functional Scale;^40^ 7-day accelerometry^41^ (using the GENEActiv which is waterproof, can be continuously worn and programmed to start and stop automatically, see http://www.geneactiv.co.uk); the Stroke Self-efficacy Questionnaire,^42^ physical exercise levels assessed by physical activity diary, the Fatigue Assessment Scale,^43^ ^44^ exercise beliefs and exercise self-efficacy questionnaires,^45^ the SF-12,^46^ the EQ-5D-5L^47^ and Stroke Quality of Life questionnaires^48^ ^49^ and the Carer Burden Index.^50^ Health and social service use will be measured through a Service Receipt Inventory.^51^ The PROMs and instructions for their completion will be collated into a booklet.

Baseline assessments will be done by a member of the research team. Physical outcome follow-up assessments (at 6 and 9 months) will be conducted at the participant's home, by a blinded assessor trained in all elements of the assessment procedure. Self-report questionnaires for 6-month and 9-month follow-ups will be compiled into a single booklet with instructions for completion, and mailed to participants along with the accelerometer and a physical activity diary, a week before the home visit, unless participants request a visit from a member of the research team to help fit the accelerometer. If materials are posted, prior to each blinded assessor visit a member of the research team will call the participant to check that they are still available to meet. During this call the member of the research team will check how the participant is progressing with the questionnaire and check whether the accelerometer is still being worn and is comfortable. Participants will also be asked to report any adverse events, enquiring explicitly about whether they have experienced any fatigue, muscle soreness or falls (all expected adverse events). If materials are delivered by a member of the research team, the accelerometer will be fitted, adverse event report taken and questionnaire booklets left for completion. The blinded assessor will check questionnaires for completeness and understanding during their visit. Table 1 indicates the outcome instruments and the time points for their use.

**Table 1:** Trial outcome measures and when used (1=baseline; 2=postintervention; 3=follow-up)

Measure	Time to administer	Assessment		
	(mins)	1	2	3
*Primary*				
Rivermead Mobility index^37^ ^38^ 15-item, dichotomously scored measure of mobility disability. 14 items are self-report and 1 (standing for 10 s without aids) is scored by observation.	5	✓	✓	✓
Timed Up and Go Test^39^ Objective measure of mobility, balance and locomotor performance, in which the individual is observed and timed rising from a chair, walking 3 m, turning and returning to the chair	5	✓	✓	✓
Modified Patient Specific Functional Scale^40^* Identification by individual of up to 5 functional tasks that are important and difficult to perform, and rating of ability to perform each task on a 0–10 scale	10	✓	✓	✓
Physical Activity Diary* Participants record the type of activity and its duration each day of the week (1–2 min/day to complete)	10–15	✓	✓	✓
Physical activity—7-day accelerometry^41^ Worn by individual to assess physical activity behaviour over 7 days. Should take 5 min to fit watch and 10 min to post back	15	✓	✓	✓
*Secondary*				
Fatigue Assessment Scale^43^ ^44^* 10-item self-completion questionnaire in which aspects of fatigue are rated on how regularly they are experienced, using a 5-point scale	5	✓	✓	✓
Stroke Self-efficacy Questionnaire^42^* 10-item questionnaire in which participants rate their confidence in completing some tasks that may have been difficult for them since their stroke	5	✓		✓
Exercise beliefs questionnaire^45^* Measures attitudes to exercise by rating levels of agreement to 5 statements about what it can achieve for the individual	3	✓		✓
Exercise self-efficacy questionnaire^45^* Self-rating of confidence to overcome 4 personal barriers to exercise	3	✓		✓
Stroke Quality of Life^48^ ^49^* Self-rating of 12 dimensions of lifestyle and personal functioning	5	✓		✓
EQ-5D-5L^47^* Measuring health-related quality of life and can be used for cost-utility analysis	3	✓		✓
SF-12^46^* Abbreviated version of the Short-Form-36 self-completion questionnaire measuring health-related quality of life. It can also be used to calculate the SF-6D, which may be used for cost-utility analysis	5	✓		✓
Service Receipt Inventory^51^* Record of types and amount of use of health and social care resources including medication, clinical contacts, formal and informal social care. Completed by assessor drawing on participant and family accounts, and clinical records if available	10	✓		✓
Carer Burden Index^50^* Carers of stroke survivors rate the difficulties and challenges of providing care	5	✓		✓
Adverse incidents^52^	3		✓	✓

*Questionnaire may be mailed for self-completion before home visit or left after a research team visit.

##### Feasibility, acceptability and process outcomes

The feasibility of a definitive RCT will be determined by collecting and analysing the following pilot study data. The numbers and details of those approached; the recruitment and retention rates, as well as recruitment patterns from each route and geographical area. Those who decline to participate or drop-out of the study will be invited to participate in a brief phone interview regarding their reasons. Acceptability of randomisation, outcome measurement burden, interventions and other aspects of trial participation will be investigated by checking completion of questionnaires and objective assessments as well as through interviews with 10 intervention and 10 control group members (purposively selected to ensure inclusion of different genders, ages, disability levels and previous exercise experience) and by postintervention interviews with the trainers. These qualitative interviews will use a semistructured interview schedule designed to cover the above issues as well as ask about other personal and contextual factors that may affect participation and outcomes (ie, barriers and facilitators) including what refinements might be needed for the definitive trial delivery. Interviews will be conducted by a member of the research team at a convenient time and location for the participant after the intervention period. Adverse events will be identified,^52^ via trainer reports and the research staff explicitly questioning participants, using trial standard operating procedures. No serious adverse events were reported in the preliminary studies. Intervention fidelity will be assessed by several methods: attendance, accelerometry, exercise diaries, session checklists and video analysis of selected training sessions in each programme.

#### Usual care

Participants in intervention and control arms may receive health and social care as part of their usual care, and these will be recorded using the Service Receipt Inventory.

#### Foreseen difficulties

(1) ‘Exercise’ as a term may be off-putting to some people. Our SUG and preliminary studies participants strongly advised that we emphasise the potential functional and quality of life benefits and not promote ReTrain as an exercise programme; however, it was agreed that community leisure centres were an acceptable venue. (2) Timing: some of those who give consent to take part may have to wait until the next ReTrain programme is ready to start. The wait will be minimised by running successive Exeter-based programmes alongside the South Devon programme and the study team will maintain brief but regular contact with those waiting. The feasibility and acceptability of this process, including the wait time and recruitment/retention issues more generally, are part of what is being tested in this pilot study. (3) Participant burden: particularly completion of the battery of measures and travel to intervention venue. These were deemed acceptable by most participants in our preliminary studies and this was affirmed during our SUG events when planning this proposal. Participant carers may experience some burden as they may need to support the participants by providing travel to and from the 23 training sessions at the community centre venue. The carer will also be asked to complete a questionnaire at two time points during the research. (4) Trainer availability: we worked with five trainers during our preliminary studies and there are currently more than 100 accredited ARNI trainers in the UK, indicating there would be capacity to deliver a future multicentre trial but more trainers might be needed if wider future implementation was indicated. Three local trainers have agreed to deliver the current study ReTrain programmes.

#### Service user involvement

Stroke survivors, their partners and carers have been consulted at all stages of the work leading to this proposal. In 5 SUG events and 14 research development meetings over 5 years our patient and public involvement (PPI) representatives have materially influenced decisions on the study population, promotion and recruitment, the nature of the ReTrain intervention and how its effectiveness should be assessed. One experienced PPI representative will be in the Trial Management Group (TMG), and another, who has experience of ARNI training, will be in the Study Advisory Group (SAG). The founder of ARNI, Dr Tom Balchin will also be part of the SAG. Our service users will also continue to contribute throughout this pilot study in terms of reviewing documentation for ethics approval, commenting on and proof reading reports and contributing to dissemination activities. They have, and will continue to be, supported in their work by the Peninsula's Collaboration for Leadership in Applied Health Research and Care (PenCLAHRC) PPI team, for example, by attending workshops on critical appraisal skills.

#### Data analysis

Given the feasibility objectives of this pilot study, the focus of data analysis will be descriptive. For both recruitment settings (Exeter and South Devon) participant flow will be summarised using the CONSORT flow diagram, reporting recruitment and attrition rates (both treatment and study drop-outs) with 95% CIs. The diagram will also reflect the number of recruitment letters sent, numbers consenting, number randomised, number undertaking intervention and number of completed outcomes alongside means and SDs regarding the number, length and frequency of sessions. All protocol deviations, along with reasons and number of missing items on questionnaires will be reported. Mean and SDs (or equivalent) for all clinical outcomes for both study arms will be reported at baseline, 6 and 9 months follow-up. For the trial process evaluation, we will use a thematic analysis for the qualitative interview data and use several of the quantitative measures (including demographic, medical and questionnaire data) to help identify and understand potential mediators and moderators of trial outcome. For the analysis of intervention fidelity, we will use the procedures we designed and tested in our preliminary studies for the video analysis combined with trainer interview data and the session checklists (that will be part of the Intervention Delivery manual). These data collection and analysis methods will be assessed for their potential to inform the process evaluation component of a definitive trial. In such a future trial, the health economics analysis will be a cost-utility analysis, using QALYs and secondary analyses will investigate the benefits of the intervention more broadly within the framework of a cost consequences approach, so offering the potential to weight different outcomes in a multicriteria decision analysis framework.^51^ In this pilot study, we will assess the relative benefits of calculating health-related quality of life using SF-6D developed from the SF-12 over the QALY calculated using EQ-5D-5L; explore the psychometric properties of the SF-12 and investigate whether it has greater ability to discriminate between the milder health states. We will assess costs associated with intervention delivery as well as assess the feasibility of collecting health and social service resource use through a Service Receipt Inventory. Any missing data in the Inventory will be recorded and we will check if any resource use categories are misunderstood by matching Inventory completion with medical records for a subgroup of participants. This will help us develop strategies to minimise missing data in a future definitive trial.

#### Study timeline

The timetable for the research can be seen in table 2: months 0–4 set up and trainer briefing; months 0–9 recruitment: including participant identification (0–9 months), screening (2–9 months), consent and baseline assessments (3–9 months); months 5–15 training programmes inclusive of 6 and 9 month follow-ups; month 18 complete the 9-month follow-ups; months 11–18 interviews; months 16–21 analysis and final reports. Trial management meetings and SUG meetings will be held on 10 occasions across the 21 months of the study.

**Table 2:** Gannt chart of study timeline

Months	0	1	2	3	4	5	6	7	8	8	9	10	11	12	13	14	15	16	17	18	19	20	21
Set up; brief trainers																							
Recruitment																							
Training programmes×3 including 6 months follow-up																							
Interviews																							
Analysis and reporting																							

#### Incentives and payments

Incentive payments will also be made to control and intervention group participants and paid on two occasions: £10 in vouchers at 6 months and £10 in vouchers at 9 months. These incentives are intended to maximise the chances of obtaining a full data set, including the views of any people who leave the intervention or control groups (but do not fully withdraw from the study) and this will assist the process evaluation. Participants have to remain in the study but do not have to complete all assessments in order to receive the vouchers.

The latest NHS Health Research Authority guidance on Payments and Incentives in Research (May 2014, Section 9.1) states that pro-rata payments based on the amount of time completed and/or the number of research procedures undertaken are legitimate. Our intention would be to disburse 50% of the incentive payment at the time of the postintervention (6 months) outcome assessment and the remainder at the time of the follow-up (9 month) assessment.

## Ethics and dissemination

### Ethics

Research and Development (R&D) approval was granted from the mid-Devon and Torbay Primary Care Trusts (1602209). The study will be conducted in accordance with the principles of the International Conference for Harmonisation of Good Clinical Practice (ICH GCP) guidelines^53^ and the Research Governance Framework for Health and Social Care.^54^ Any amendments to the trial documents will be approved by the sponsor before submission to the Research Ethics Committee (REC) and R&D departments. This is a University of Exeter-sponsored research study, working in collaboration with NHS trusts. The University's Clinical Trials insurance cover provides either legal liability cover or non-negligent/no-fault compensation cover. Research passports and letters of access will be acquired for the Trial Manager and Associate Research Fellow so that they may visit GP surgeries to extract data during medical notes review.

#### Governance and safety considerations

Drafts of the trial protocol have been reviewed by the Southwest Research Design Service and the Stroke Clinical Studies Group. The trial is registered with the ClinicalTrials.gov: trial number NCT02429180. The study sponsor is the University of Exeter.

##### Adverse events

In preliminary studies, several falls were recorded, though none required medical intervention. Transitory muscle soreness and high levels of postexercise fatigue were also reported in some cases, but there appears to be a low risk of serious adverse advents associated with this intervention. Those providing the intervention are trained in health and safety procedures and will be required to ask participants about any adverse events occurring at home, and record any reported or witnessed during the intervention. Participants will also be asked about adverse events as part of the process evaluation (to maintain blinding of the outcomes assessor). Any serious adverse event will be immediately reported to the trial sponsor and relevant ethics committee if the chief investigator (CI) deems it related to the intervention, and to the independent Trial Steering Committee (TSC) members who are also acting as our Data Monitoring and Ethics Committee.

#### Trial monitoring and management

Day-to-day running of the trial will be the responsibility of the trial manager. Standard operating procedures will be written for: (1) assessment processes and reporting; (2) data management; (3) adverse incidents monitoring, reporting and action; (4) study staff health and safety.

The core study team (CI, trial manager and assistant research fellow) will meet at least fortnightly during the study. TSC meetings will be held (if necessary by teleconference) on three occasions. The TSC will discuss recruitment, withdrawals, study progress, process evaluation and adverse events, and will advise on protocol amendments where necessary. The steering group will include academics with expertise in trial methodology, health economics, qualitative methods and process evaluation, clinicians with expertise in stroke and rehabilitation, and stroke survivors. Owing to the low risk of adverse events, an independent Data Monitoring and Safety Committee will not be appointed for this pilot study. Instead, we will appoint suitably qualified academics and clinicians to the TSC who will have responsibility for independently monitoring the safety and quality of the trial. A closed meeting prior to the TSC meetings will take place with the independent members of the TSC who will also be responsible for oversight of the safety of the trial and data integrity (thereby taking on the role of the Data Monitoring and Ethics Committee).

Our preliminary studies suggest that the trial does not pose any specific risks to individual participants nor does it raise any substantial ethical issues. Based on results from our development work ReTrain is a low-risk intervention. Participants will be informed of possible benefits and known risks of participation in the trial by means of a patient information leaflet and discussion with the research team. All participants will sign a consent form approved by the Ethics Committee. They will be consented to participating in the trial, being randomised and followed up, for accessing their medical records to review their primary and secondary care resource use, for video-recording of training sessions, participating in and being audio-recorded during interview and being contacted in the future about this and other research. Individuals who are not able or not willing to be randomised will not be recruited. Individuals will be sent an additional patient information leaflet relating to the interview study. Written consent will be obtained again immediately prior to the interview study, the video-recorded training sessions, and prior to medical notes review. This will be performed to allow the participants to fully consider their participation decisions and to confirm or change their original consenting decision.

##### Data management

Data will be collected and retained in accordance with the UK Data Protection Act 1998, and managed in accordance with the trial-specific standard operating procedure for data management. With their consent, participant details will be passed between the NHS and the research team by telephone or in person (ie, not electronically via email or text message). All enrolled participants will be allocated a unique study ID, and the information linking their ID to their personal details will be kept securely at the University of Exeter. All other participant-related paper records will be anonymised and stored separately from the personal information. The electronic database for the trial will be stored on the secure servers of the University of Exeter with password-controlled access provided for the research team by the Peninsula Clinical Trials Resource Unit. Single data entry with extensive in-built validity checks will be used to reduce the risk of transcription errors. The study database will include prompts for missing data, and warnings to alert staff when values are entered that are outside of the expected range or are inconsistent with other data already entered, or if the type of value entered is incorrect (eg, a numeric value entered rather than text).

Video-recordings and audio-recordings will be digitised, encrypted and stored on the university secure server. Audio-recordings will be retained until after anonymised transcripts have been finalised and analysed. At this stage they will be securely and permanently deleted. Access to personal data will be restricted to the research team. Names and participant details will not be passed onto any third parties and no named individuals will be included in the write up of the results.

All participants (stroke survivors and personal trainers) will be asked for their consent for the study team to retain interviews and video-recordings for the purposes of future research by those involved directly in the study team or for educational purposes.

All study data will be kept for 10 years under secure conditions on University of Exeter secure servers. Data will also be subject to standard secure storage and usage policies.

### Dissemination and impact activities

Trial progress will be reported to our SUG quarterly. At the end of the study, we will seek input from our SUG to help disseminate a lay summary of the findings to study participants. A trial publication policy will be developed. We envisage a number of key papers arising from this pilot trial. The publication policy document will outline the strategic plan for dissemination. The results of the trial will be reported first to study collaborators and to the funder (the Stroke Association). The sponsor and funder play no role in the study design, conduct, analyses, data interpretation or report writing. The funder requires advance notification of any planned public dissemination activities but does not hold authority over these activities. The main report will be drafted by the TMG and circulated to all collaborators for comment. The final version will be agreed by the TSC before submission for publication, on behalf of all the ReTrain collaborators. We will report to our objectives and hold meetings with our TSC, TMG and SUG to discuss whether we have a sufficient case to apply for funds to run a definitive RCT of ReTrain.

Key outputs from the trial will contribute to our dissemination and impact agenda: (1) pilot data will inform the decision whether to proceed to a definitive RCT and if so we will have (2) the evidence to support a funding application and (3) finalised Trainer and Intervention Delivery manuals to enable multicentre replication of ReTrain; (4) presentations at national and international conferences, seminars and PPI events and (5) dissemination through internationally recognised peer-reviewed journal publications (including open access web sources), newsletters and media releases.
